# *POU* genes are expressed during the formation of individual ganglia of the cephalopod central nervous system

**DOI:** 10.1186/2041-9139-5-41

**Published:** 2014-11-05

**Authors:** Tim Wollesen, Carmel McDougall, Bernard M Degnan, Andreas Wanninger

**Affiliations:** Department of Integrative Zoology, Faculty of Sciences, University of Vienna, Althanstr. 14, 1090 Vienna, Austria; School of Biological Sciences, The University of Queensland, Brisbane, QLD 4072 Australia

**Keywords:** brain, complex, evolution, development, homeobox genes, invertebrate, Lophotrochozoa, mollusk, ontogeny

## Abstract

**Background:**

Among the Lophotrochozoa, cephalopods possess the highest degree of central nervous system (CNS) centralization and complexity. Although the anatomy of the developing cephalopod CNS has been investigated, the developmental mechanisms underlying brain development and evolution are unknown. *POU* genes encode key transcription factors controlling nervous system development in a range of bilaterian species, including lophotrochozoans. In this study, we investigate the expression of *POU* genes during early development of the pygmy squid *Idiosepius notoides* and make comparisons with other bilaterians to reveal whether these genes have conserved or divergent roles during CNS development in this species.

**Results:**

*POU2*, *POU3*, *POU4* and *POU6* orthologs were identified in transcriptomes derived from developmental stages and adult brain tissue of *I. notoides*. All four *POU* gene orthologs are expressed in different spatiotemporal combinations in the early embryo. *Ino-POU2* is expressed in the gills and the palliovisceral, pedal, and optic ganglia of stage 19 to 20 embryos, whereas the cerebral and palliovisceral ganglia express *Ino-POU3. Ino-POU4* is expressed in the optic and palliovisceral ganglia and the arms/intrabrachial ganglia of stage 19 to 20 individuals. *Ino-POU6* is expressed in the palliovisceral ganglia during early development. In stage 25 embryos expression domains include the intrabrachial ganglia (*Ino-POU3*) and the pedal ganglia (*Ino-POU6*). All four *POU* genes are strongly expressed in large areas of the brain of stage 24 to 26 individuals. Expression could not be detected in late prehatching embryos (approximately stage 27 to 30).

**Conclusions:**

The expression of four *POU* genes in unique spatiotemporal combinations during early neurogenesis and sensory organ development of *I. notoides* suggests that they fulfill distinct tasks during early brain development. Comparisons with other bilaterian species reveal that *POU* gene expression is associated with anteriormost neural structures, even between animals for which these structures are unlikely to be homologous. Within lophotrochozoans, *POU3* and *POU4* are the only two genes that have been comparatively investigated. Their expression patterns are broadly similar, indicating that the increased complexity of the cephalopod brain is likely due to other unknown factors.

## Background

**Figure 1:**
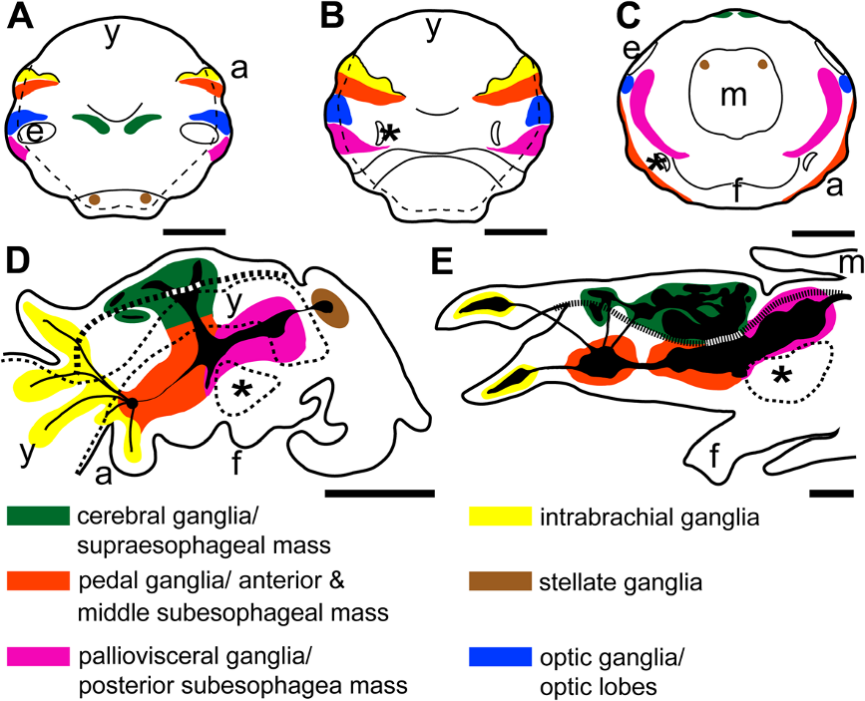
**Sketch drawings highlighting the development of individual ganglia into a centralized brain in** ***I. notoides*** **.** In stages older than stage 19, the neuropil (black) consolidates in the center of various brain lobes. **(A)** Dorsal view of a stage 19 embryo (anterior faces up). **(B)** Ventral view of a stage 19 embryo (anterior faces up). **(C)** Posterior view of a stage 19 embryo (dorsal faces up). **(D)** Sagittal section along the midline of a stage 23 embryo (anterior faces to the left). **(E)** Sagittal section along the midline of the anterior body regions of a stage 30 hatchling (anterior faces to the left). The esophagus is marked by a thick dashed line and the internal and external yolk portions are outlined by a thinner dashed line in D and E. The statocysts are marked by asterisks. Abbreviations: a, arm; e, eye; f, funnel; m, mantle; y, yolk. Modified from [8, 12]. Scale bars 150 μm.

Cephalopod mollusks such as squids, cuttlefish, octopuses, and nautiluses are one of the most fascinating invertebrate groups with respect to their cognitive abilities and behavioral repertoire [1–3]. Although coleoid cephalopods, that is, all cephalopods except for nautiluses, exhibit a number of molluscan plesiomorphies, they also possess unique (autapomorphic) features such as a highly centralized CNS (Figure 1), which is composed of more than 35 individual brain lobes in some species. Intricately connected to sensory and motor systems, the CNS is arranged around the esophagus with perikarya surrounding the neuropil (Figure 1; [4]). The adult brains of *Octopus vulgaris* and *Loligo vulgaris* belong to the invertebrate brains that have been best investigated by means of classical histological techniques and investigations on the developing brains commenced in the 1970s (*L. vulgaris*[5]; *O. vulgaris*[6]; *Sepioteuthis lessoniana*[7]; *Idiosepius paradoxus*[8]; *Nautilus pompilius*[9], reviewed in [10]). Only recently, the spatiotemporal distribution of certain neuronal markers was investigated in detail for a limited number of cephalopod species. These studies revealed that selected neuropeptides and neurotransmitters are expressed during early CNS development and thus appear to play a role during early neurogenesis [11–16]. They also suggest that certain neuronal populations that contain these substances might be homologous among coleoids [12, 13, 16].

Although coleoid cephalopods are ideal systems to investigate the molecular underpinnings of the development of complex invertebrate brains, little is known about genes that regulate the establishment of the cephalopod CNS. One group of genes that is involved in neurogenesis and that is well studied for ecdysozoan and vertebrate model systems (but less so for lophotrochozoans, except for a few isolated accounts on gastropod mollusks and the annelid model *Platynereis dumerilii*) is the *POU* gene family. *POU* genes are a metazoan-specific class of homeobox genes that encode transcription factors [17, 18]. Detailed studies on model organisms such as fruit fly, frog, mouse, or rat suggested that these genes orchestrate the proper spatiotemporal expression of a plethora of genes involved in crucial developmental processes (Table 1, [19, 20]). While *POU* gene orthologs are consistently expressed in the nervous system of adults and developmental stages of a number of phylogenetically distant taxa such as acoels, cnidarians, insects, tunicates and fishes (Table 1; [19]), expression domains also comprise other epithelia such as the cephalochordate ectodermal sensory cells and gill slits, the pronephridial duct of the zebrafish, and the statocysts and gonads of hydrozoans (Table 1). Functional studies suggest that *POU* genes are employed in gastrulation and apoptosis in vertebrates as well as in segmentation and specification of neural precursor cells in the fruit fly [19]. In contrast, only sparse information is available on *POU* gene expression in the Lophotrochozoa. Although few studies identified *POU* genes as being expressed in various tissues by means of RT-PCR or immunohistochemistry [21–23], studies by *in situ* hybridization on the spatiotemporal expression in lophotrochozoans only exist on the gastropod *Haliotis asinina* (*POU3* and *POU4*) and the annelid *P. dumerilii* (*POU4*) (Table 1; [24–27]). In larval developmental stages of *H. asinina, POU3* is expressed in the anlagen of the CNS but also in non-neural domains such as mucus cells of the foot, a portion of the visceral mass, the statocysts, and the anlagen of the radular sac (Table 1; [25]). In adults, expression domains comprise the cerebral and pleuropedal ganglia, epipodial tentacles, tentacles, eyes, gills, and muscles (Table 1; [24]). *POU4* is expressed in different sets of ectodermal cells in the foot, in the vicinity of the esophageal ganglia, and in the statocysts of the larvae of *H. asinina* (Table 1; [26]). Moreover, *POU4* is expressed in cells in the mantle, in cells in the vicinity of the eyes and the ctenidial and osphradial anlagen of developmental stages of *H. asinina*. In adults, *POU4* expression domains resemble those of *POU3* and include the cerebral and pleuropedal ganglia, the epipodial tentacles, eyes, tentacles, and gills (Table 1; [24]). In the annelid *P. dumerilii POU4* (*Brn3*) is expressed in the neuroectoderm of the ventral nerve cord and probably in neurons of the parapodial ganglia (Table 1; [27]). Interestingly, a recent EST analysis of stage 16 to 28 specimens of the cuttlefish *Sepia officinalis* did not reveal *POU* gene transcripts [28]. The authors suggested that, besides possible technical reasons, *POU* genes might solely be expressed in earlier developmental stages, that is, stages that do not yet exhibit anlagen of the nervous system ([28], cf. [11, 29] for staging of specimens).

**Table 1:** **Metazoan** ***POU*** **gene expression domains with focus on lophotrochozoan taxa as revealed by** ***in situ*** **hybridization experiments**

SUPER-PHYLUM/phylum species	Expression domains in developmental stages (D) or adults (A)	Reference
Acoela		
*Neochildia fusca*	A: (*BRN-1*): stem neoblasts?, neurons, intraepidermal gland cells	[30]
	A: *POU4* (*BRN-3*): neurons	
Cnidaria		
*Craspedacusta sowerbyi*	A: *POU4f1*: bell margin in statocysts between tentacles	[31]
	A: *POU4f2*: bell margin in statocysts between tentacles, gonads	
	A: *POU4f3*: close to center of bell quadrants, gastric cavity	
	A: *POU6*: statocysts, gonads	
*Aurelia* spec.	A: *POU1* (*PIT1*): rhopalia	[32]
LOPHOTROCHOZOA		
Gastropoda		
*H. asinina*	D: trochophore: *POU3*: 2 bilateral ectodermal (mucus) cells in centroposterior foot anlage, two cells in anterolateral foot anlage	[24–26]
	Pre-torsional veliger: 2 bilateral ectodermal (mucus) cells in centroposterior foot (close to operculum)	
	Post-torsional veliger: 2 bilateral ectodermal (mucus) cells in centroposterior foot (close to operculum), pleuropedal, cerebral, esophageal ganglia, branchial ganglia, dorsoposterior region of visceral mass, statocysts, radular sac anlage	
	A: *POU3*: cerebral and pleuropedal ganglia, epipodial fringe, tentacle, eye, gill, muscle	
	D: trochophore: *POU4*: single cell in prospective mantle edge of trochophore larva), bilateral pair of ventral ectodermal cells in anterocentral region of foot anlage +2 additional cells later	
	Pre-torsional veliger: anterocentral ectoderm of foot, no expression in mantle, additional pair of cells in lateromedian ectoderm of foot	
	Post-torsional veliger: ventral ectoderm of foot + lateral expansion of anterocentral cells, cells in vicinity of prospective eyes, 2 territories on left side of cephalopedal and visceropallial junction (vicinity of esophageal ganglia), cells close to mouth, statocysts, vicinity of ctenidial and osphradial anlagen	
	A: *POU4*: cerebral and pleuropedal ganglia, epipodial fringe, eye, tentacle, gill	
Annelida		
*P. dumerilii*	A: *POU4* (*BRN3*): expression in longitudinal columns which are segmentally clustered along regenerated ventral nerve cord and in cells in developing parapodia (parapodial ganglia?)	[27]
ECDYSOZOA		
*Drosophila melanogaster*	D: *POU2* (*PDM-1* (*POU-19*), *PDM-2* (*POU-29*)): neuroectoderm, (peripheral) sensory organs	[19, 33–35]
	D: *POU3* (*CF1a*): ectodermal segmental expression, tracheal cells, mesectodermal cells arranged along longitudinal ventral midline of embryo	
	D: *POU4* (*I-POU*): supraesophageal ganglia and ventral nerve cord	
*Caenorhabditis elegans*	D: *POU2* (*CEH-18*): muscles and epidermis	[36–38]
	A: gonadal sheath cells	
	D: *POU3* (*CEH-6*): neurons	
	D: *POU4* (*UNC-86*): neural precursor cells	
	A: neurons	
DEUTEROSTOMIA		
*Ciona intestinalis* (Tunicata)	D: embryo: *POU4*: neural precursor cells in PNS. Restricted to posterior sensory vesicle and motoneurons of visceral ganglion in CNS	[39]
*Branchiostoma floridae* (Cephalochordata)	D: embryo and larva: *POU4*: anteriormost neural plate and in bilateral ectodermal (sensory?) cells of neurula. Subsequently, expression in motoneurons behind posterior cerebral vesicle and in segmentally arranged motoneurons in hindbrain, in rostrum and epidermal sensory cells close to mouth.	[40]
*Branchiostoma floridae* (Cephalochordata)	D: embryo and larva: *POU3*: expression in dorsal epiblast and entire neural plate except a portion close to cerebral vesicle. Expression in primordium of gill slits, pharynx and left Hatschek´s diverticulum.	[41]
*Danio rerio* (Vertebrata)^a^	D: embryo: *POU3*: expression in fore-, mid-, and hindbrain, in spinal cord and pronephric duct	[42]
*Xenopus laevis*	D: *POU1*: neural fold stage: anterior neural plate; tailbud stage: anterodorsal portion (eye and brain); A: skin and brain	[43, 44]
	D: *POU2*: neurula stage: anterior nerve cord; tailbud stage: anterodosal region; A: adults in kidney and brain	
	D: *POU3*: neurula stage: brain and spinal cord, auditory vesicle	[45]
*Rattus norvegicus*	A: *POU1* (*PIT-1*), *POU2* (*OCT-2*), *POU3* (*BRN-2*), *POU4* (*BRN-3*), *TST-1*, *OCT-1*: nervous system	[33, 46]
	D: *POU6* (*BRN-5*): developing CNS, spinal cord	
	A: brain, kidney, lung, heart, testis, pituitary	

^a^See [19] for an exhaustive list on *POU* gene expression domains in developing and adult vertebrates.

In order to facilitate a comparative approach, the transcriptomes of whole animals of prehatching developmental stages as well as of the isolated adult CNS of the pygmy squid *I. notoides* were screened for candidate *POU* orthologs. In this study, expression patterns of *Ino-POU2*, *Ino-POU3*, *Ino-POU4*, and *Ino-POU6* are described for developmental stages of the pygmy squid *I. notoides* to determine where *POU* genes are expressed during the development of a coleoid cephalopod. This work represents the most comprehensive study on *POU* genes in lophotrochozoans and offers insights into their expression in an invertebrate with a highly centralized and complex brain.

## Methods

### Collection, RNA extraction, and fixation of animals

Adults of the pygmy squid *I. notoides* were dip-netted in the seagrass beds of Moreton Bay, Queensland, Australia. Embryos were cultured and staged as described previously [12, 47]. After the removal of egg jelly and chorion, the RNA of approximately 300 specimens covering developmental stages from the freshly laid zygotes to post-hatching individuals, that is, stages 0 to 30 and hatchlings [47], was extracted using TriReagent according to the manufacturer’s instructions (Astral Scientific Pty. Ltd., Caringbah, Australia). For adult squids, RNA was extracted from the entire CNS of seven adults. Stage 19 to 30 individuals were fixed for *in situ* hybridization experiments (see [12, 47] for staging criteria).

### RNAseq and transcriptome assembly

RNA of developmental stages was sequenced by 454 technology; RNA retrieved from the entire adult central nervous system was sequenced by Illumina technology (Eurofins, Ebersberg, Germany). A total of 588,878 reads with an average read length of 377 bp were obtained from the 454 sequencing. These reads were subsequently filtered (rRNA removal), and adapter and low quality sequences were trimmed, normalized, and assembled *de novo* by Eurofins. The 38,267,214 Illumina reads (100-bp long, paired-end) were filtered (rRNA removal), and adapter and low quality sequences trimmed, normalized, and assembled *de novo* into contigs with the assembler Trinity [48] by the authors. The 454 transcriptome comprises 55,555 contigs (N50 = 620), whereas the Illumina transcriptome comprises 166,289 contigs (N50 = 977).

### Alignment and phylogenetic analysis

Known amino acid sequences of bilaterian *POU* gene orthologs retrieved from NCBI were used in BLAST searches against the assembled transcriptomes. Amino acid sequences were aligned and the conserved motifs of the POU specific domain and the POU-type homeodomain were used to reconstruct trees, which were generated using Jukes-Cantor as Genetic Distance Model and Neighbor-Joining as Tree build Method implemented in the program Geneious Pro 5.5.6 (Biomatters, Auckland, New Zealand, http://www.geneious.com).

### Molecular isolation of RNA transcripts

First-strand cDNA was synthesized by reverse transcription of RNA pooled from different developmental stages using the First strand cDNA Synthesis Kit for rt-PCR (Roche Diagnostics GmbH, Mannheim, Germany). Gene-specific primers were designed from identified *POU* genes and transcripts were amplified via standard PCR. PCR products were size-fractioned by gel electrophoresis, and bands of the expected length were excised. Gel bands were cleaned up using a QIAquick Gel Extraction Kit (Qiagen, Hilden, Germany). Cleaned-up products were cloned by insertion into pGEM-T Easy Vectors (Promega, Mannheim, Germany) and plasmid minipreps were grown overnight. Finally, plasmids were sent for sequencing, and orthologs of *Ino-POU2*, *Ino-POU3*, *Ino-POU4*, and *Ino-POU6* were identified using the BLASTx algorithm screening the database of the National Center for Biotechnology Information (NCBI).

### Probe syntheses and whole-mount *in situ*hybridization

The probe template was amplified from the miniprepped plasmids via standard PCR using M13 forward and reverse primers, and *in vitro* transcription reactions were performed with these templates, digoxigenin-UTP (DIG RNA Labeling Kit, Roche Diagnostics), and SP6 polymerase (Roche Diagnostics GmbH) for the syntheses of antisense riboprobes, according to the manufacturer’s instructions. Whole-mount *in situ* hybridization experiments were carried out as described previously [12]. Briefly, developmental stages were rehydrated into PBT (PBS +0.1% Tween-20). They were treated with Proteinase-K (20 mg/ml in PBT at 37°C for 15 min) and prehybridized in hybridization buffer for 4 h at 55 to 65°C. Hybridization with a probe concentration of 0.5 to 1 μg/ml was carried out overnight at 55 to 65°C. For each gene, a minimum of 20 individuals per stage was investigated. In addition, negative controls were carried out with sense probes for all genes and developmental stages. After hybridization, selected developmental stages were embedded in a solution of gelatin-ovalbumin, vibratome-sectioned, and mounted on glass slides in order to facilitate the identification of expression domains in the individual brain lobes. In addition, the majority of whole-mount preparations were cleared in a solution of benzyl-benzoate and benzyl alcohol, analyzed, and the results were documented with a Nikon Eclipse E800 microscope. If necessary, images were processed with Adobe Photoshop 9.0.2 software (San Jose, CA, USA) to adjust contrast and brightness.

### Statement of ethical approval

Animals were collected, anesthetized, and fixed according to internationally recognized standards (University of Queensland Animal Welfare Permit No. 158/09 ‘The cultivation of *Idiosepius* (pygmy squid) for studies in developmental biology’ to BMD).

## Results

### *POU*gene orthologs and phylogenetic analysis

**Figure 2:**
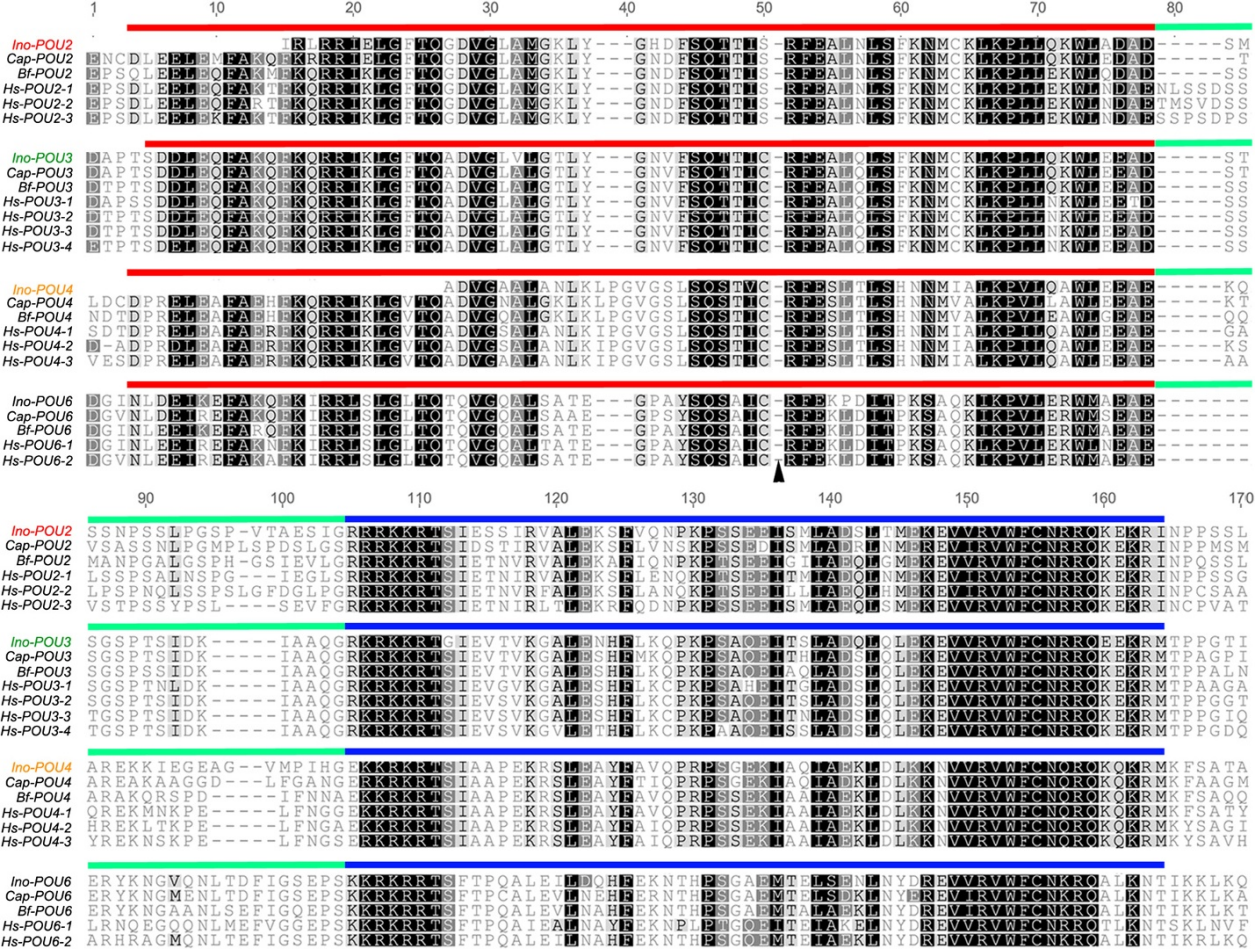
**Alignment of deduced amino acid sequences of Ino-POU2, Ino-POU3, Ino-POU4, and Ino-POU6 compared to selected bilaterian orthologs.** Residues colored in black are identical, dark gray-colored ones are 80 to 100% conserved and white ones less than 60% conserved. The POU-domain comprises an N-terminal POU specific domain (red) and a C-terminal POU-type homeodomain (blue), which are connected via a linker region (green). The 36 amino acids of the sequence Hs-POU6-2 have been deleted, as indicated by an arrowhead. GenBank accession numbers of amino acid sequences used for alignment: *I. notoides*: Ino-POU2: [GenBank:KM892881], Ino-POU3: [GenBank:KM892882], Ino-POU4: [GenBank:KM892883], Ino-POU6: [GenBank:KM892884]; *Branchiostoma floridae*: Bf- POU1: ABP01321.1, Bf- POU2: XP_002610677.1, Bf-POU3: AAL85498.1, Bf- POU4: ABC42926.1, Bf- POU6: XP_002608796.1; *Capitella teleta*: Cap-POU2: ELU09478.1, Cap-POU3: ELU03597.1, Cap-POU4: ELT93355.1, Cap-POU6: ELU13951.1, *Homo sapiens*: Hs-POU2 isoform 1: NP_002688.3, Hs-POU2 isoform 2: NP_002689.1, Hs-POU2 isoform 3: NP_055167.2, Hs-POU3 isoform 1: NP_002690.3, Hs-POU3 isoform 2: NP_005595.2, Hs-POU3 isoform 3:, NP_006227.1, Hs-POU3 isoform 4:, NP_000298.3, Hs-POU4 isoform 1: NP_006228.3, Hs-POU4 isoform 2: NP_004566.2, Hs-POU4 isoform 3: NP_002691.1, Hs-POU6 isoform 1: NP_002693.3, Hs-POU6 isoform 2: NP_009183.3.

**Figure 3:**
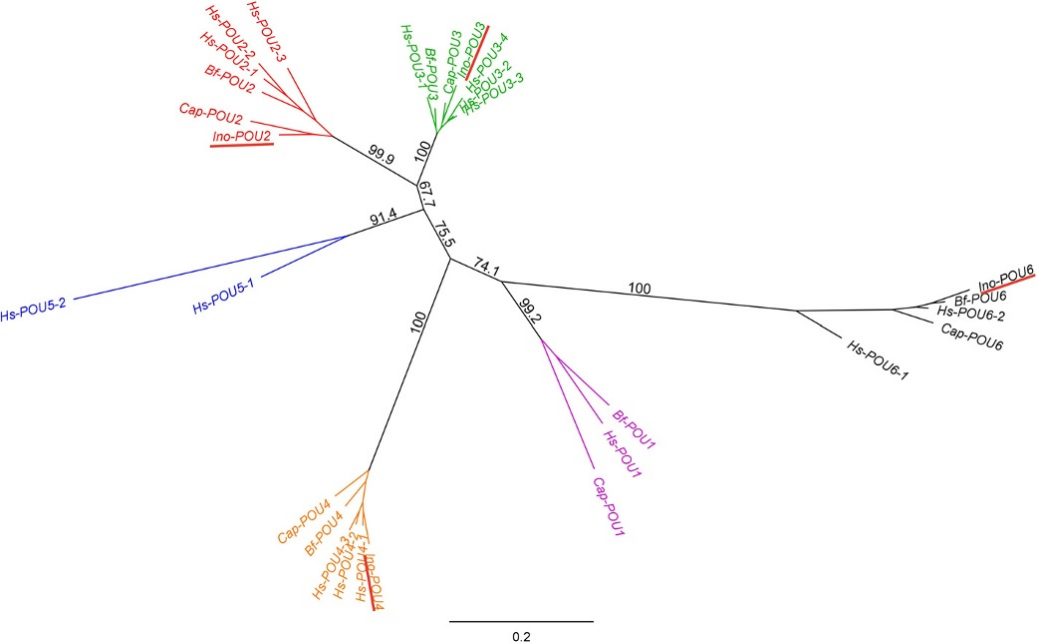
**Neighbor-joining consensus tree of the POU gene family including deduced amino acid sequences of POU1 (pink), POU2 (red), POU3 (green), POU4 (orange), POU5 (blue), and POU6 (black) of** ***I. notoides*** **and selected bilaterians.** All subfamilies are robustly supported. Tree nodes are supported by bootstrap values of 1,000 replicates and the consensus support (%). Branch length of the tree represents amino acid substitution per site (see scale bar for measurement). GenBank accession numbers of all sequences are itemized in caption of Figure 2 or mentioned as follows: *Capitella telata*: Cap-POU1: ELT90027.1, Cap-POU5: ELT90025.1; *Branchiostoma floridae*: Bf-POU1: ABP01321.1; *Homo sapiens*: Hs-POU1: NP_000297.1; Hs-POU5 isoform 1: NP_002692.2, Hs-POU5 isoform 2: NP_694948.1.

Both *I. notoides* developmental and brain transcriptomes contain assembled transcripts encoding four POU homeodomain transcription factors of varying lengths. Ino-POU2, Ino-POU3, Ino-POU4 and Ino-POU6 were recovered as partial sequences of 158, 190, 139 and 163 amino acids (aa) in length, respectively (Figure 2). The coding sequences comprise an N-terminal POU-specific domain and a C-terminal POU-type homeodomain, which are separated by a linker region (Figure 2). All four Ino-POU amino acid sequences cluster with their bilaterian orthologs, as revealed by our phylogenetic analysis (Figure 3).

### Overall anatomy and ontogeny of the central nervous system in *I. notoides*

The ontogeny of the CNS of *I. notoides* and its sister species *I. paradoxus* was described previously [8, 12]. In stage 17 to 18 embryos, neuroblasts ingress, migrate from the ectoderm, and coalesce to establish the anlagen of the future paired cerebral, pedal, palliovisceral, optic, stellate, and intrabrachial ganglia during early development, when the ectodermal layer covers the yolk syncytium (Figure 1A, B and C; [8, 12]). Successively, the individual ganglia develop neuropilar regions with the palliovisceral ganglia exhibiting the most prominent neuropil during early development (Figure 1D). Traditionally, all ganglia are named ‘lobes’ after stage 23. At this stage, the palliovisceral ganglia develop into the posterior subesophageal and the periesophageal mass (Figure 1D and E), the pedal ganglia develop into the anterior and middle subesophageal mass, and the supraesophageal mass develops from the cerebral ganglia. These masses comprise various individual lobes (up to 35 in some coleoid cephalopods), which are subject to increased neuropilar growth and a relative decrease of perikarya during subsequent development. The adult CNS of *I. notoides* resembles that of its sister species *I. paradoxus*[8, 12, 13, 49]. It is composed of a central brain and two laterally attached optic lobes that process the visual stimuli from the laterally attached eyes. The brain is composed of a supraesophageal and a subesophageal mass, which are divided by the esophagus and are laterally connected with each other (Figure 1). The periesophageal mass is located ventrally to the posterior subesophageal mass and is often difficult to distinguish from the middle and posterior subesophageal mass shown in Figure 1E.

### *POU*gene expression

Gene expression patterns of stage 19 to 30 (hatchlings) individuals were analyzed, and the stages in which expression patterns change are presented.

### *Ino-POU2*expression

**Figure 4:**
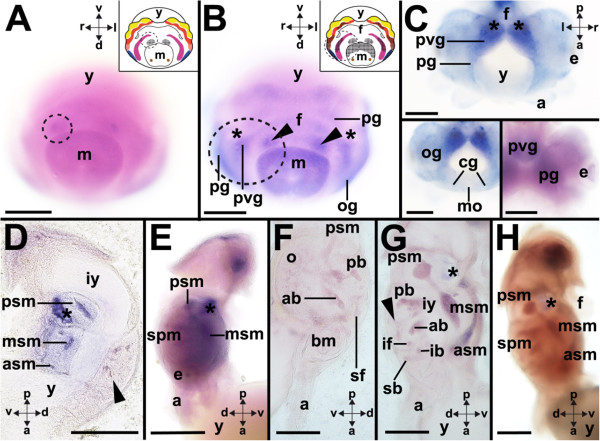
***Ino-POU2*** **-expression during development of** ***I. notoides*** **.** Cartoons highlight *Ino-POU2*-expression as black checkered domains (see Figure 1 for color-code of ganglia). Dorsal (d)-ventral (v), anterior (a)-posterior (p), and left (l)-right (r) axes indicate the orientation. **(A)** During stage 19 *Ino-POU2* is expressed in the gills close to the mantle (m). **(B)** Stage 20 embryos exhibit expression in the gills (arrowheads), the posterior mantle, the palliovisceral (pvg), the pedal (pg), and the optic ganglia (og) (posteroventral view). **(C)** Stage 21 individuals globally express *Ino-POU2* including the palliovisceral and pedal ganglia, however not in the cerebral ganglia (cg) or the funnel (upper and lower left inset). *Ino-POU2* is expressed in the eyes (e) during stage 22 (lower right inset) (yolk sac removed in images). **(D)** During stage 24, *Ino-POU2* is expressed in the anterior (asm), middle (msm), and posterior subesophageal masses (psm). Slight expression was observed in the lobes of the supraesophageal mass (arrowhead) (sagittal vibratome section). The staining in the statocyst is unspecific. **(E)** During stage 25, *Ino-POU2* is expressed in the brain including the supraesophageal mass (spm). **(F)** Close-up of *Ino-POU2* expression in the supraesophageal mass during stage 26. Expression domains comprise the anterior (ab) and posterior basal lobes (pb), the superior frontal lobe (sf), and the optic lobes (o) (sagittal vibratome section). **(G)** The expression domains of later embryos resemble the ones of stage 26 individuals (sagittal vibratome section). During stage 27 *Ino-POU2* is expressed in the vertical lobe complex (arrowhead) of the supraesophageal mass. *Ino-POU2* is expressed in the superior buccal (sb) and inferior buccal lobes (ib) as well as the inferior frontal lobes (if). **(H)** *Ino-POU2* expression during stage 27. Asterisks mark the statocysts. Abbreviations: a, arm; f, funnel; iy, internal yolk; mo, mouth; y, yolk. Scale bars: A-E: 150 μm, F-H: 200 μm.

**Figure 5:**
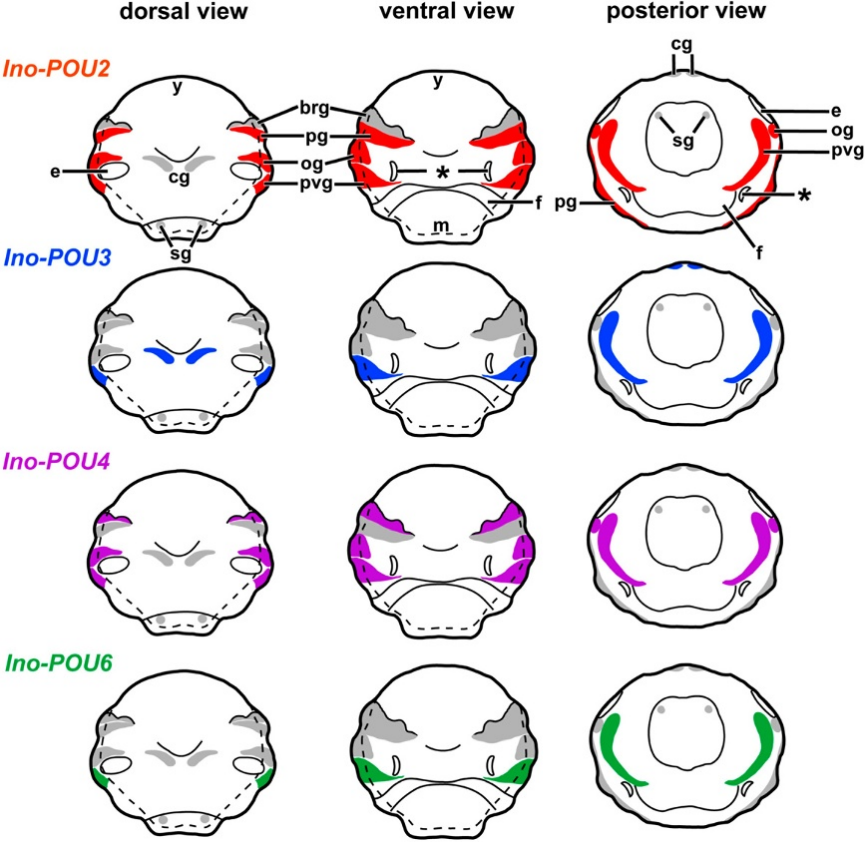
**Expression of** ***Ino-POU2*** **(orange),** ***Ino-POU3*** **(blue),** ***Ino-POU4*** **(pink), and** ***Ino-POU6*** **(green) in stage 19 to 20 embryos of** ***I. notoides*** **.** Anlagen of ganglia that do not express a given *POU* gene are labeled in gray. Abbreviations: cg, cerebral ganglion; e, eye; f, funnel; brg, intrabrachial ganglion; m, mantle; sg, stellate ganglion; og, optic ganglion; pg, pedal ganglion; pvg, palliovisceral ganglion; y, yolk. The statocysts are labeled with asterisks. Modified from [8, 12].

Stage 19 embryos express *Ino-POU2* in the anlagen of the gills (Figures 4A and 5). In subsequent developmental stages *Ino-POU2* is also expressed in the palliovisceral, pedal, and optic ganglia as well as in the posterior mantle (Figure 4B). Expression gains in intensity in stages 21 to 22 individuals, while a diffuse global expression was observed in the animal with exception of the arms (Figure 4C). The strongest *Ino-POU2*-expression was observed in the palliovisceral and pedal ganglia and the eyes (Figure 4C). This situation remains similar in stage 24 individuals, whose anterior, middle and posterior subesophageal masses express *Ino-POU2*, while a few individual lobes of the supraesophageal mass exhibit weak staining (Figure 4D). This changes in stage 25 individuals, which strongly express *Ino-POU2* throughout the brain and also at a lower level in the optic lobes (Figure 4E). In stage 25 individuals, and subsequent developmental stages, *Ino-POU2* is expressed in the supraesophageal, periesophageal, and subesophageal masses (Figure 4E,F,G and H). In the supraesophageal mass, the anterior basal and posterior basal lobes express *Ino-POU2* (Figure 4F,G). Lobes of the vertical lobe complex, such as the superior frontal lobe and the subvertical lobe, also express *Ino-POU2* (Figure 4G). The optic lobes also exhibit slight expression of *Ino-POU2* (Figure 4F). *Ino-POU2*-expression domains of stage 27 individuals resemble those of stage 26 individuals (Figure 4H); however, later prehatching developmental stages cease to express *Ino-POU2*.

### *Ino-POU3*expression

**Figure 6:**
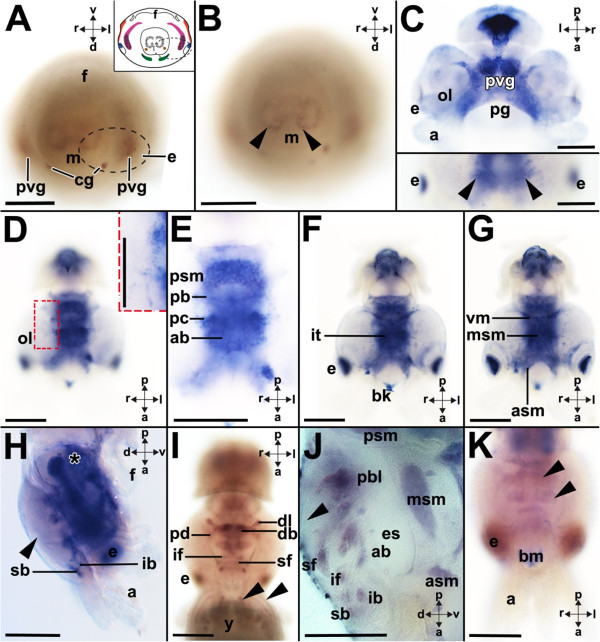
***Ino-POU3*** **-expression during development of** ***I. notoides*** **.** The cartoon highlights *Ino-POU3*-expression as black checkered domains (see Figure 1 for color-coded ganglia). Dorsal (d)-ventral (v), anterior (a)-posterior (p), and left (l)-right (r) axes indicate the orientation. Staining around shell gland is unspecific (C, D, F, G). **(A)** During stage 19 *Ino-POU3* is expressed in the cerebral (cg) and palliovisceral ganglia (pvg). **(B)** Same specimen as shown in A expressing *Ino-POU3* in the mantle (arrowheads). **(C)** Stage 22 individuals express *Ino-POU3* in the pedal (pg) and palliovisceral ganglia, the eyes (e), and the anterior basal lobes (arrowheads in lower inset). D-G: Optical sections from dorsal to ventral of a stage 24 individual (unspecific staining in the beak (bk)). **(D)** *Ino-POU3* is expressed in the brain including the peduncle lobe (arrowhead in inset). **(E)** Close-up of the supraesophageal and posterior subesophageal masses (psm). The anterior basal (ab), posterior basal (pb), and precommissural lobes (pc) express *Ino-POU3*. **(F)** The interbasal lobes (it) connect to the subesophageal mass and express *Ino-POU3*. **(G)** *Ino-POU3*-expression in the anterior (asm) and middle subesophageal masses (msm) and in the ventral magnocellular lobes (vm). **(H)** *Ino-POU3*-expression in the superior (sb) and inferior buccal lobes (ib) but not in the vertical lobe complex (arrowhead) during stage 25. The statocysts are labeled with an asterisk. **(I)** During stage 25 *Ino-POU3* is expressed in the intrabrachial ganglia (arrowheads), the dorso-lateral (dl), the peduncle (pd), the superior frontal (sf) and inferior frontal lobes (if). **(J)** During stage 26 *Ino-POU3* is expressed in the brain including the inferior buccal (ib) and superior buccal lobes (sb) but not the vertical and subvertical lobes (arrowhead). **(K)** Weak *Ino-POU3*-expression during stage 27. Abbreviations: a, arm; bm, buccal mass; es, esophagus; f, funnel; m, mantle; ol, optic lobe. Scale bars: A-G: 150 μm, H-K: 200 μm.

Stage 19 embryos express *Ino-POU3* in the distal portion of both cerebral ganglia and the anterior palliovisceral ganglia (Figures 6A and 5). In addition, *Ino-POU3* expression is present in two circular domains in the mantle (Figure 6B). Expression in the palliovisceral ganglia gains in intensity in subsequent developmental stages such as stage 22, and further expression is present in the pedal ganglia and the eyes (Figure 6C). The first supraesophageal expression domain appears slightly later in what are probably the anlagen of the anterior basal lobes in the cerebral ganglia (Figure 6C, lower inset). The expression domains widen during stage 24 when *Ino-POU3* expression is also present in large portions of the supraesophageal mass (Figure 6D,E). The posterior subesophageal mass, periesophageal mass and the peduncle lobes express *Ino-POU3*, in contrast to both optic lobes (Figure 6D,E). *Ino-POU3* is expressed in the anterior and posterior portion of the anterior basal lobes (Figure 6E), the precommissural lobes, and the lobes of the posterior basal lobes (Figure 6E). Further expression was observed in lobes that connect to the middle subesophageal mass such as the interbasal lobes (Figure 6F). The ventral magnocellular lobes (one of the three periesophageal lobes) as well as the middle and anterior subesophageal masses also express *Ino-POU3* (Figure 6G). *Ino-POU3*-expression is stronger in subsequent developmental stages such as stage 25 individuals and apparent in the superior and inferior buccal lobes (Figure 6H) but also the superior and inferior frontal lobe (Figure 6I). Additional lobes that express *Ino-POU3* are the dorso-lateral, dorsal basal, and the peduncle lobes (Figure 6I). The intrabrachial lobes of the arms also express *Ino-POU3* at stage 25 (Figure 6I), but not in other developmental stages (c.f. for example, Figure 6K). *Ino-POU3* is expressed at a higher level in stage 26 individuals; however, no expression is found in the subvertical and vertical lobes (arrowhead in Figure 6J). Stage 26 to 30 individuals do not exhibit *Ino-POU3* expression in the eyes (Figure 6K). Stage 27 individuals and older prehatching stages exhibit only weak or no *Ino-POU3*-expression in their brains (Figure 6K). Besides a few *Ino-POU3* expressing neurons (Figure 6D) the optic lobes do not express *Ino-POU3* (for example, Figure 6I,K).

### *Ino-POU4*expression

**Figure 7:**
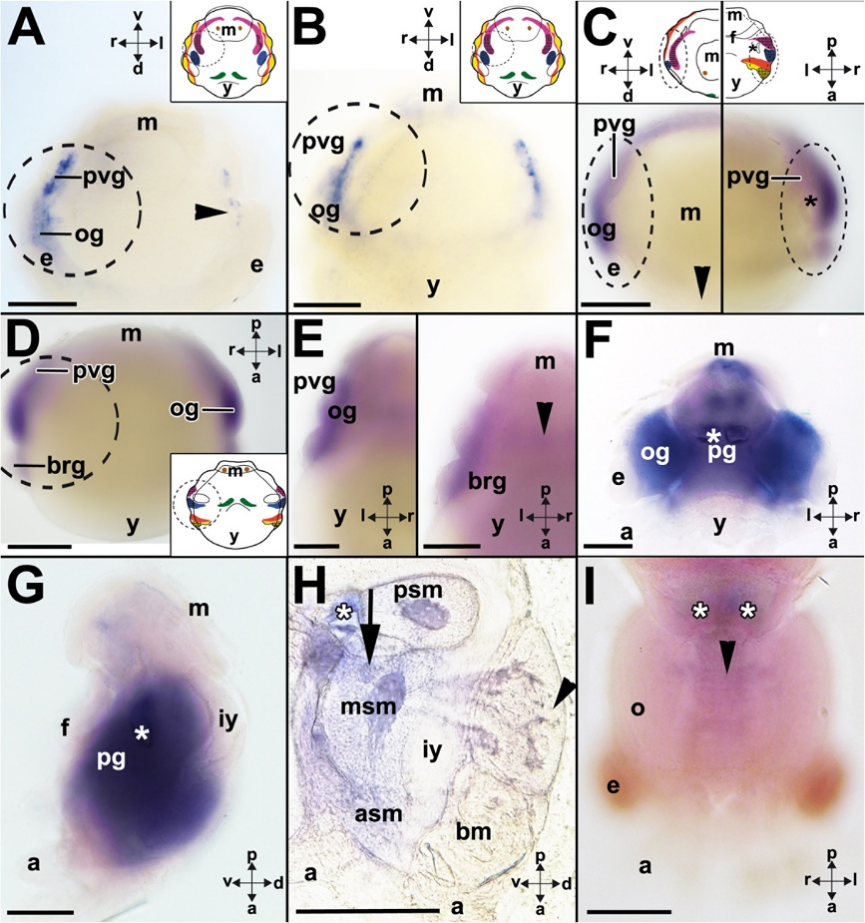
***Ino-POU4*** **-expression during development of** ***I. notoides*** **.** Cartoons highlight *Ino-POU4*-expression as black checkered domains (see Figure 1 for color-code of ganglia). Dorsal (d)-ventral (v), anterior (a)-posterior (p), and left (l)-right (r) axes indicate the orientation. **(A)** Stage 19 individuals exhibit *Ino-POU4*-expression in the palliovisceral (pvg) and optic ganglia (og) close to the eyes (e) (dorsoposterior view). In this specimen, *Ino-POU4* staining is weaker on the left side (arrowhead) than on the right side. **(B)** During stage 19 *Ino-POU4* is expressed in the palliovisceral and optic ganglia (dorsoposterior view). **(C)** Stage 20 individuals exhibit *Ino-POU4*-expression in the optic and palliovisceral ganglia (dorsoposterior view). Note the absence of *Ino-POU4* expression in the cerebral ganglia (arrowhead). **(D)** The optic, palliovisceral, and intrabrachial ganglia (brg) of stage 20 individuals express *Ino-POU4*. **(E)** Stage 21 individuals exhibit *Ino-POU4* expression in the palliovisceral and optic ganglia (left inset), the intrabrachial and the pedal ganglia (arrowhead) (right inset). **(F)** Stage 22 individuals exhibit strong *Ino-POU4*-expression in the optic ganglia as well as in the palliovisceral and pedal ganglia (pg). No *Ino-POU4* is expressed in the intrabrachial ganglia of the arms (a). **(G)** The *Ino-POU4*-expression pattern of stage 23 individuals resembles that of stage 22 individuals. **(H)** Vibratome section along the midline of the cephalic region during stage 25. Note the lack of *Ino-POU4*-expression in the vertical lobe (arrowhead). Besides the anterior (asm), middle (msm), and posterior subesophageal (psm) and supraesophageal mass, also the ventral magnocellular lobes (arrow) express *Ino-POU4*. **(I)** Stage 27 individuals exhibit only faint *Ino-POU4*-expression in the posterior basal lobes (arrowhead) and expression patterns of subsequent developmental stages resembles the one of stage 27 specimens. Abbreviations: bm, buccal mass; f, funnel; iy, internal yolk; m, mantle; y, yolk. The statocysts are marked by asterisks. Scale bars: A-G: 150 μm, H-I: 200 μm.

*Ino-POU4* is expressed in the optic and palliovisceral ganglia of stage 19 individuals (Figures 7A,B, and 5). In some individuals, *Ino-POU4* is expressed more weakly on the left or right side of the body (arrowhead in Figure 7A). Subsequent developmental stages such as stage 20 individuals show increased expression of *Ino-POU4* in the optic and palliovisceral ganglia (Figure 7C) and also in the anlagen of the arms, that is, in the intrabrachial ganglia (Figure 7D). Stage 21 individuals exhibit slight expression of *Ino-POU4* in the pedal ganglia (arrowhead in right inset of Figure 7E). Stage 22 and 23 specimens exhibit strong *Ino-POU4* expression in the optic ganglia, as well as in the palliovisceral, pedal, and cerebral ganglia (Figure 7F, G). Expression in the intrabrachial ganglia could no longer be observed (Figure 7F, G). In stage 25 and 26 individuals, expression of *Ino-POU4* is extended to large parts of the CNS (Figure 7H). Expression domains include all lobes of the anterior, middle, and posterior subesophageal masses (Figure 7H). In addition, the lobes of the periesophageal mass and the lobes of the supraesophageal mass with exception of the vertical lobe express *Ino-POU4* (Figure 7H). Subsequent developmental stages until hatchlings exhibit only faint or no *Ino-POU4*-expression in the CNS (Figure 7I).

### *Ino-POU6*expression

**Figure 8:**
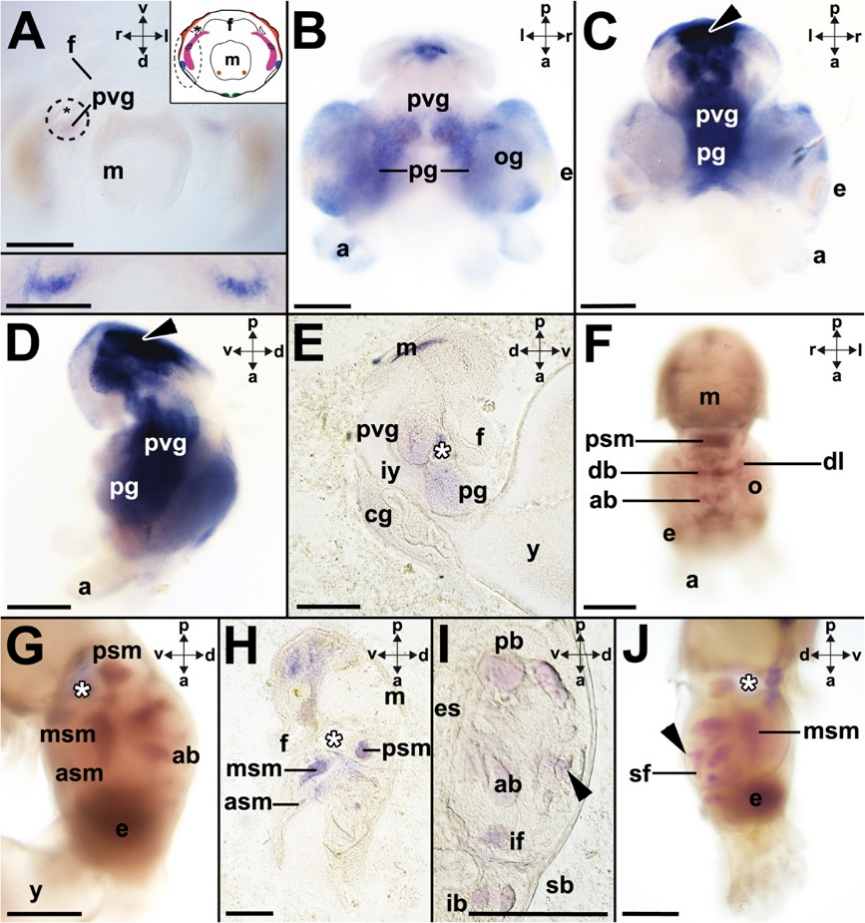
***Ino-POU6*** **-expression during development of** ***I. notoides*** **.** Cartoons highlight *Ino-POU6*-expression as black checkered domains (see Figure 1 for color-coded ganglia). Dorsal (d)-ventral (v), anterior (a)-posterior (p), and left (l)-right (r) axes indicate the orientation. **(A)** *Ino-POU6*-expression in the palliovisceral ganglia (pvg) during stage 19. Inset: Close-up of both palliovisceral ganglia. **(B)** *Ino-POU6*–expression in the pedal (pg) and palliovisceral ganglia during stage 22. **(C)** During stage 23 *Ino-POU6* is expressed in the pedal and palliovisceral ganglia (unspecific staining around shell gland (arrowhead)). **(D)** *Ino-POU6*–expression in the pedal and palliovisceral ganglia during stage 23 (unspecific staining around shell gland (arrowhead)). **(E)** Vibratome section along the midline of a stage 23 individual with *Ino-POU6* being expressed in the pedal and palliovisceral ganglia but not in the cerebral ganglia (cg). **(F)** During stage 25 *Ino-POU6* is expressed in the posterior subesophageal mass (psm), the anterior basal (ab) and posterior basal lobes including the dorso-lateral (dl) and dorsal basal lobes (db) but not the optic lobes (o). **(G)** During stage 25 *Ino-POU6* is expressed in the brain including the anterior (asm), middle (msm), and posterior subesophageal masses. **(H)** *Ino-POU6*-expression during stage 26 resembles that observed during stage 25 (vibratome section). **(I)** *Ino-POU6*-expression in the supraesophageal mass during stage 26 including the subvertical (arrowhead), the anterior basal (ab) and posterior basal lobes (pb), the inferior frontal (if), the inferior buccal (ib) and the superior buccal lobes (sb) (vibratome section). **(J)** During stage 27 *Ino-POU6* is expressed among others in the subvertical lobe (arrowhead) and superior frontal lobe (sf). Subsequent prehatching stages cease to express *Ino-POU6* in their CNS. Abbreviations: a, arm; e, eye; f, funnel; iy, internal yolk; m, mantle; og, optic ganglion; y, yolk. The statocysts are labeled by asterisks. Scale bars: A-E and I: 150 μm, F-H and J: 200 μm.

In early developmental stages such as stage 19, *Ino-POU6* is expressed in the palliovisceral ganglia (Figures 8A and 5). In stage 22 individuals, *Ino-POU6* is additionally expressed in the pedal ganglia (Figure 8B). Subsequent developmental stages, such as stage 23, show strong expression in their pedal and palliovisceral ganglia (Figure 8C,D). Faint or no expression is present in the supraesophageal mass of stage 23 individuals (Figure 8E). Stage 25, 26, and stage 27 individuals are characterized by strong expression of *Ino-POU6* in large parts of the CNS (Figure 8F, G, H, I and J). Lobes of the anterior, middle, and posterior subesophageal masses and the periesophageal mass exhibit strong expression of *Ino-POU6* (Figure 8F, G), including the anterior basal and posterior basal lobes (Figure 8F, G) and the dorso-lateral and dorsal basal lobes of the supraesophageal mass (Figure 8F). The subvertical and superior frontal lobes of the vertical lobe complex also express *Ino-POU6* (Figure 8I, J). Weak or no *Ino-POU6*-expression was detected in late prehatching developmental stages (not shown).

## Discussion

### Four *POU*genes are expressed during *I. notoides*development

In this study, four *POU* gene orthologs were identified in the transcriptomes derived from early developmental stages and adult CNS tissue of the pygmy squid *I. notoides*. The amino acid sequences of Ino-POU2, Ino-POU3, Ino-POU4 and Ino-POU6 cluster with other bilaterian POU orthologs (Figure 3) and are expressed in early developmental stages as revealed by *in situ* hybridization experiments (Figures 4, 6, 7, 8 and 5). This is in contrast to a recent EST analysis on developmental stages of the cuttlefish *S. officinalis*, which did not detect *POU* gene transcripts [28]. Given our findings and those in other studies investigating *POU* gene expression in mollusks, the lack of *POU* gene orthologs in the *S. officinalis* EST library may be a technical artifact rather than an actual absence of these transcripts [23, 25, 26, 28]. To date, orthologs of the remaining two *POU* subfamilies, *POU1* and *POU5*, have not been reported from any lophotrochozoan. *POU1* appears to be ancestral while *POU5* might be a vertebrate innovation [18, 50].

### Comparative expression of *I. notoides POU*genes

*Ino-POU2*-expression is mainly restricted to the anlagen of the gills; the posterior mantle; and the pedal, palliovisceral and optic ganglia (Figures 4A, B and 5). Besides these expression domains, *Ino-POU2* is also expressed globally in the embryo (Figure 4B,C,E). To date, no *POU2*-expression patterns are known for any other lophotrochozoan (Table 1). The genes *PDM-1* (*POU-19*) and *PDM-2* (*POU-29*) of *Drosophila melanogaster* possess high sequence similarity to other bilaterian *POU2*-orthologs and are expressed in the neuroectoderm but also in peripheral sensory organs (Table 1). In *I. notoides POU2* is only expressed in few sensory epithelia, that is, the eyes of stage 22 individuals (Figure 4C). *CEH-18*, the nematode *POU2* ortholog, is expressed in muscles and the epidermis of developmental stages and in the gonadal sheath cells of adults (Table 1). In vertebrates such as the frog *Xenopus laevis* and the rat *Rattus norvegicus POU2* orthologs are expressed in the anterior brain region during development (Table 1). This is in contrast to *I. notoides* with *POU2* being expressed in the pedal and palliovisceral ganglia, which are located in the posterior region of the CNS (present study). Early expression in both these ganglia suggests that *POU2* is involved in the establishment of these brain regions. Stage 25 to 27 individuals express *Ino-POU2*-throughout the CNS, an expression pattern that resembles patterns of all other *POU* genes in these late prehatching developmental stages.

Although there is only limited information on the expression of *POU* genes in lophotrochozoan taxa, *POU3*-expression has been documented for a vetigastropod, the tropical abalone *H. asinina*[24, 25]. In the abalone trochophore, *Has-POU3* transcripts are present in two large posterior cells (possibly mucus cells) and two smaller antero-lateral cells in the anlage of the foot ectoderm [25]. In *I. notoides*, the first *POU3*-expressing cells are located in the anlagen of the cerebral and palliovisceral ganglia of stage 19 individuals (Figures 6A and 5). In addition, two circular expression domains, which are not associated with the shell gland, are located in the embryonic mantle ectoderm (Figure 6B). Post-torsional veligers of *H. asinina* are the first to express *Has-POU3* in all ganglia of the developing adult CNS, that is, the pleuropedal, cerebral, esophageal, and branchial ganglia [25]. A more gradual increase of *POU3*-expression can be observed in the ganglia and brain lobes of *I. notoides*. Stage 22 individuals first express *Ino-POU3* in the palliovisceral and pedal ganglia and subsequently in increasing domains of the supraesophageal brain lobes. *POU3* expression in the cerebral ganglia of *H. asinina* and the supraesophageal mass of *I. notoides* supports the traditional view that these brain regions are homologous [51]. Homology has also been claimed for the gastropod pedal ganglia and the cephalopod subesophageal mass, which each express *POU3. H. asinina,* as well as *I. notoides,* express *POU3* in their cephalic appendages, that is, the gastropod tentacles [24] and the cephalopod intrabrachial ganglia of stage 25 individuals (arrowheads in Figure 6I). Notably, *Ino-POU3*-expression in the CNS ceases in the late prehatching stages of *I. notoides* (c.f. Figure 6J,K), resembling the condition in *H. asinina* where no *Has-POU3*-transcripts were observed in the CNS but in were observed in different expression domains such as the dorsoposterior visceral mass, the presumptive anlagen of the radula sac, and the statocysts [25]. Adult abalone express *POU3* in their cerebral and pleuropedal ganglia but also the epipodial tentacles, the tentacles, eyes, gills, and muscles [24]. Stage 22 individuals of *I. notoides* also strongly express *POU3* in their eyes (Figure 6C). In adult *I. notoides, POU3*, as well as all other identified *POU* genes, are expressed in brain tissue as revealed by transcriptome screens; however, the individual expression levels are unknown.

*Ino-POU4* is predominantly expressed in the CNS during early development of *I. notoides*. The first expression domains are the anlagen of the palliovisceral and optic ganglia (Figures 7A,B and 5). Subsequently, the arms also express *Ino-POU4*. It has been proposed that *POU4* orthologs may play a role during the differentiation process of distinct populations of sensory cells in a variety of bilaterians (Table 1; [19, 39]). In *I. notoides* the only *Ino-POU4* expression domain that bears vast numbers of sensory cells are the anlagen of the arms, which cease to express *Ino-POU4* during subsequent development (cf. Figure 7D,G). Post-torsional larvae of *H. asinina* also express *Has-POU4* in both developing cephalic tentacles [26]. In *H. asinina*, few cell somata express *Has-POU4* in the presumptive anlage of the central posterior foot of the trochophore and the anterior central foot anlage of the veliger larva, regions with high abundances of chemo- and mechanoreceptors [26]. Post-torsional animals retain expression in the ventral ectoderm of the foot but also express *Has-POU4* in presumptive anlagen of the eyes and in the vicinity of the esophageal ganglia [26]. In *I. notoides Ino-POU4* transcripts have not been located in the eyes but in the optic ganglia of stage 19 to 23 individuals (Figure 7A,B,C,D,E and F). Other sensory expression domains comprise the statocysts and the ctenidial and osphradial rudiment in late veliger larvae of the gastropod *H. asinina*[26]. In *P. dumerilii, POU4* is expressed in cells of the developing parapodia, probably belonging to the parapodial ganglia (Table 1, [27]). These findings demonstrate that at least *POU3* and *POU4* are involved in the formation of the peripheral nervous system of the lophotrochozoan species investigated.

During early development *Ino-POU6* is first expressed in the palliovisceral ganglia (Figure 5). In subsequent developmental stages, expression extends to the pedal ganglia and finally the supraesophageal mass (Figures 8 and 5). Although *POU6*-orthologs are known from other protostome invertebrates, no expression patterns have been published so far for lophotrochozoan representatives (Table 1). The adult hydrozoan *C. sowerbyi* expresses *POU6* in the statocysts and gonads (Table 1; [31]). *BRN-5*, the *POU6* ortholog of the rat, is intensely expressed in the developing CNS and spinal cord; however adults express *BRN-5* in the kidney, lungs, heart, testis, pituitary gland as well as the brain (Table 1; [33, 46]). As stated for all other *Ino-POU* genes, *Ino-POU6* expression levels decrease in late prehatching individuals (that is, stage 27 to 30). This resembles the situation in the developing rat brain in which transcript levels of *POU6* decrease from embryonic day 15 to postnatal day 10 [46].

### The role of *POU*genes in CNS development

A major similarity between the CNS of animals as different as rodents, fruit flies, and pygmy squids is the expression of *POU* genes in the anteriormost brain region [33], (present study). The fruit fly´s protocerebrum + deuterocerebrum, the murine telencephalon + diencephalon + mesencephalon, and the gastropod and cephalopod cerebral ganglia express *POU* genes but lack anterior *HOX* gene expression (Table 1; [33, 52–56]). This indicates that in these bilaterian representatives, *POU* transcription factors are involved in the development of the anteriormost neural territories, which are unlikely to be homologous according to anatomical and ontogenetic evidence and currently accepted phylogenies [57]. However, more gene expression patterns of basal representatives of various bilaterian clades, as well as expression patterns of potential bilaterian sister groups such as acoels or cnidarians, are needed to assess their role during development [57, 58].

The present study demonstrates that at least four *POU* genes are expressed during early cephalopod CNS development and in the adult CNS. All major ganglia exhibit unique expression patterns in early embryos, indicating different roles during the establishment of these brain regions (Figure 5). In subsequent prehatching developmental stages all four *POU* genes are intensely expressed in wide parts of the brain; however, expression of all *POU* genes ceases in late prehatching embryos (approximately stage 27 to 30). Interestingly, only few of the investigated *POU* genes are expressed in the optic lobes, and peripheral ganglia such as the intrabrachial ganglia only express few *POU* orthologs in a small developmental time frame. Further expression analyses on *POU* genes and other homeobox genes in other molluscan representatives will also contribute to our knowledge on the role of these genes during development in lophotrochozoans.

## Conclusions

All four *Ino-POU* genes are expressed in unique spatiotemporal combinations during early neurogenesis, which indicates that they are involved in distinct processes during early brain development. As reported for other phylogenetically distantly related bilaterians, expression is associated with anteriormost neural structures, which are unlikely to be homologous. *POU3* and *POU4* are the only *POU* genes within lophotrochozoans that have been comparatively studied. Since their expression patterns are broadly similar, the increased complexity of the cephalopod brain might be due to other unknown factors.

## Abbreviations

a: arm
a: anterior (in the orientation axes)
aa: amino acid
ab: anterior basal lobe
asm: anterior subesophageal mass
bk: beak
BLAST: basic local alignment search tool
bm: buccal mass
bp: base pairs
brg: intrabrachial ganglion
cDNA: complementary deoxyribonucleic acid
cg: cerebral ganglion
CNS: central nervous system
d: dorsal
db: dorsal basal lobe
DIG: digoxigenin-11-uridin-triphosphate
dl: dorso-lateral lobe
e: eye
es: esophagus
f: funnel
EST: expressed sequence tags
ib: inferior buccal lobe
if: inferior frontal lobe
it: interbasal lobe
iy: internal yolk
l: left
m: mantle
mo: mouth
msm: middle subesophageal mass
NCBI: national center for biotechnology information
o: optic lobe
og: optic ganglion
p: posterior
pb: posterior basal lobe
pvg: palliovisceral ganglion
PBS: phosphate buffered saline
PBT: phosphate buffered saline +0.1% TritonX
pc: precommissural lobe
pd: peduncle lobe
pg: pedal ganglion
PNS: peripheral nervous system
psm: posterior subesophageal mass
r: right
RNA: ribonucleic acid
RNAseq: ribonucleic acid sequencing
rRNA: ribosomal ribonucleic acid
RT-PCR: reverse transcription polymerase chain reaction
sb: superior buccal lobe
sf: superior frontal lobe
sg: stellate ganglion
spm: supraesophageal mass
v: ventral
vm: ventral magnocellular lobe
y: yolk.
